# Comparative analysis of health-related fitness in patients with acute versus chronic Chagas disease

**DOI:** 10.7705/biomedica.6892

**Published:** 2024-03-31

**Authors:** Clara Narcisa Silva Almeida, Ariane Cardoso Vasconcelos, Caroline da Silva Sousa, Nivea Thayanne Melo Silva, Dilma do Socorro Moraes de Souza, Carlos Alberto Marques de Carvalho, Suellen Alessandra Soares de Moraes, Laura Maria Tomazi Neves

**Affiliations:** 1 Postgraduate program in Human Movement Sciences, Institute of Health Sciences, Federal University of Pará, Belém, Pará, Brazil Universidade Federal do Pará Institute of Health Sciences Federal University of Pará Belém Pará Brazil; 2 Faculty of Physiotherapy and Occupational Therapy, Institute of Health Sciences, Federal University of Pará, Belém, Pará, Brazil Universidade Federal do Pará Institute of Health Sciences Federal University of Pará Belém Pará Brazil; 3 João de Barros Barreto University Hospital, Belém, Pará, Brazil João de Barros Barreto University Hospital Belém Pará Brazil; 4 Department of Pathology, Center for Biological and Health Sciences, Federal University of Pará, Belém, Pará, Brazil Universidade Federal do Pará Department of Pathology Center for Biological and Health Sciences Federal University of Pará Belém Pará Brazil

**Keywords:** Chagas cardiomyopathy, Chagas disease, exercise test, maximal respiratory pressures, muscle strength, musculoskeletal development, cardiomiopatía chagásica, enfermedad de Chagas, prueba de esfuerzo, presiones respiratorias máximas, fuerza muscular, desarrollo musculoesquelético

## Abstract

**Introduction.:**

Although Chagas disease causes high levels of morbidity, the muscle function and tolerance to physical activity in Chagas disease patients are still not completely understood.

**Objective.:**

To compare health-related fitness of patient groups with acute Chagas disease versus chronic Chagas disease.

**Materials and methods.:**

We conducted a cross-sectional study involving 18 patients. The data were obtained from patient's records, and functional capacity was measured with the six-minute walk test, the peripheral muscle strength with handgrip strength, and respiratory muscle strength using the maximum inspiratory pressure and the maximum expiratory pressure.

**Results.:**

The 18 patients were divided in two groups: acute Chagas disease (n=9) and chronic Chagas disease (n=9). The distance walked in the six-minute walk test was lower than the predicted distance walked in both groups (p < 0.0001). The maximum expiratory pressure was lower than the predicted one (p = 0.005), and statistically significant for chronic Chagas disease patients (p = 0.02). Heart rate increased faster in the chronic Chagas disease group within the first two minutes of the six-minute walk test (p = 0.04). The sixminute walk test in the acute Chagas disease group presented a strong correlation with peripheral muscle strength (p = 0.012) and maximum inspiratory pressure (p = 0.0142), while in the chronic Chagas disease group, only peripheral muscle strength and maximum inspiratory pressure were correlated (p = 0.0259).

**Conclusion.:**

The results suggest lowered functional capacity and reduced respiratory and peripheral muscle strength in patients with Chagas disease, although no differences were observed between groups. The early increase in heart rate during exercise in the chronic Chagas disease group implies a greater myocardial overload.

## Resumen

**Introducción.:**

Aunque la enfermedad de Chagas causa gran morbilidad, la función muscular y la tolerancia a la actividad física de estos pacientes aún no se comprenden por completo.

**Objetivos.:**

Comparar el estado físico relacionado con la salud de pacientes con enfermedad de Chagas aguda versus crónica de Chagas.

**Materiales y métodos.:**

Se hizo un estudio transversal que incluyó 18 pacientes. La información se obtuvo de los registros de los pacientes. La capacidad funcional se evaluó con la prueba de la caminata de seis minutos, se determinó la fuerza de prensión manual para evaluar los músculos periféricos y se estableció la fuerza de los músculos respiratorios mediante presión inspiratoria máxima y la presión espiratoria máxima.

**Resultados.:**

Se evaluaron 18 pacientes, nueve con enfermedad de Chagas aguda y nueve con enfermedad de Chagas crónica. La distancia recorrida en la prueba de caminata de seis minutos fue menor que la distancia recorrida predicha en ambos grupos (p < 0,0001). La presión espiratoria máxima fue más baja de lo previsto (forma aguda: p = 0,005; forma crónica: p = 0,02). La frecuencia cardíaca aumentó más rápido en el grupo con enfermedad de Chagas crónica dentro de los primeros dos minutos de la caminata (p = 0,04). La distancia recorrida en el grupo con la forma aguda se correlacionó con la fuerza de prensión manual (p = 0,01) y la presión inspiratoria máxima (p = 0,01). En el grupo con la enfermedad crónica, solo hubo correlación entre la fuerza de presión manual y la presión inspiratoria máxima (p = 0,02).

**Conclusiones.:**

Los resultados sugirieren disminución de la capacidad funcional y de la fuerza muscular respiratoria y periférica, aunque sin diferencias entre ambos grupos. El aumento de la frecuencia cardiaca en el grupo con enfermedad de Chagas crónica sugiere una mayor sobrecarga miocárdica.

Chagas disease is caused by the intracellular protozoan *Trypanosoma cruzi.* According to data from Sinan (2000 to 2013), oral transmission is currently the most frequent contagious vehicle, followed by vectorial transmission ^1^. In addition, non-traditional vectors are involved in parasite transmission, increasing concerns about its dissemination to nonendemic countries ^2^.

Due to the alarming numbers of infected individuals and deaths from cardiac involvement, the diagnosis and management of Chagas disease concomitantly with related comorbidities currently has become one of the major health challenges. The disease causes high levels of morbidity and mortality, and approximately 10 million people are affected worldwide. The latest estimates from the Pan American Health Organization show that there are approximately 8 million people with Chagas disease in Latin America and around 56,000 new cases per year, most of them originating in rural areas ^3^.

Chagas disease has an initial acute phase, which lasts from six to eight weeks, usually asymptomatic or with few specific symptoms, followed by a long latency period, where the patient does not present clinical manifestations, known as the chronic phase of the disease ^4^. During progression to the chronic phase, approximately 30% of Chagas disease patients become symptomatic and develop cardiac involvement (chronic chagasic cardiomyopathy). The most common complications are pulmonary or systemic thromboembolism, conduction disorders, bradyarrhythmias, severe ventricular arrhythmias, sudden death, and, mainly, congestive heart failure ^4^^,^^5^.

In acute Chagas disease, the protozoan leads to myocarditis with an intense inflammatory infiltrate, predominantly formed by CD4+ and CD8+ T cells. In this stage, clinical manifestations occur in less than 10% of the cases, and heart failure may occur. In chronic Chagas disease, 70% of the patients do not present symptoms, and routine exams do not reveal alterations ^6^. This condition can last for the entire life of the infected person, or the patient can evolve with cardiac manifestations (cardiomyopathy, arrhythmias) and/or digestive ones (megaesophagus, megacolon) ^7^.

Individuals with Chagas cardiomyopathy may present impaired respiratory muscle function, usually leading to progressive fatigue, dyspnea, and reductions in functional capacity, quality of life, and participation in community activities. A previous study ^8^ demonstrated the presence of respiratory muscle weakness associated with the left ventricular ejection fraction in patients with Chagas cardiomyopathy. In addition, cardiopathy patients generally exhibit muscular fatigue and dyspnea during exertion and limitations in performing daily activities ^8^. In this way, respiratory muscle weakness and physical deconditioning may be involved in increased respiratory work and poor functional performance in those patients ^9^.

Although cardiac involvement in Chagas disease is well known, data on peripheral and respiratory muscle functions are still scarce, so it is the disease phase in which functional impairment is detectable. Carrying out scientific research involving this group of patients is justified by the importance of understanding the characteristics and functional repercussions the pathology can have in the acute and chronic phases due to its impact on physical performance and quality of life ^10^.

Considering the clinical importance of the outcome of the disease, the increasing incidence of Chagas disease, especially in endemic countries, the current presence of nontraditional vectors involved in the transmission of Chagas-causing parasite, and the functional repercussions and limitations the pathology can cause, we aimed to compare cardiorespiratory functional capacity, and peripheral and respiratory muscle strength in patients with acute and chronic Chagas disease, considering the normative values of each group, and further analyzing whether there is a correlation between these parameters.

## Materials and methods

### Study design

We conducted a cross-sectional study comparing functional capacity and respiratory and peripheral muscle strength in acute and chronic Chagas disease patients.

### Participants

We recruited participants by convenience from the Multidisciplinary Laboratory of Chagas Disease of the João de Barros Barreto University Hospital. Inclusion criteria were: 1) aged between 18 and 60 years, and 2) classification of Chagas disease determined by parasitological and serological tests in symptomatic or asymptomatic patients.

We divided the patients according to the infection period. The group in the acute phase included individuals infected for two to eight weeks, with the presence of circulating parasites by peripheral blood direct examination and detection of anti- *Trypanosoma cruzi*serum IgM antibodies. In the chronic phase group, individuals had an infection for more than eight weeks and reactive serological tests (ELISA and indirect hemagglutination) with positive IgG serology. We excluded subjects with effortreactive hypertension, unstable angina, or who did not participate in the complete assessment of the study.

### Procedures

Data collection occurred between February and March 2016. We analyzed the medical records of previously selected patients and extracted data related to age, sex, occupation, origin, disease stage, locality where the patient was infected, form of contamination, and existing comorbidities. Participants underwent an assessment of functional capacity and respiratory and peripheral muscle strength. The same researcher (blinded) performed all the measurements. We carried out the assessments at the João de Barros Barreto University Hospital.

### Instruments

#### Functional capacity

The six-minute walk is a submaximal exercise test and was performed according to the norms of the American Thoracic Society ^11^. Patients walked the longest distance possible within six minutes on a 30-meter-long straight corridor to assess functional capacity. We measured heart rate and peripheral oxygen saturation with a NONIN Onyx 9500™ (China) finger pulse oximeter and systolic and diastolic blood pressure with a BR20D™ (Wenzhou Hongshun Industries & Tradeco, China) blood pressure monitor. The rate of perceived exertion was measured using the modified BORG CR10 scale ^12^ before and immediately after the test. We evaluated the rate-pressure product before and after the sixminute walk test and calculated it according to the equation described by Ansari *et al.*^13^. We estimated predicted values considering sex and age, considering the Brazilian population ^14^.

#### Respiratory muscle strength

To evaluate respiratory muscle strength, we connected a previously calibrated analogic manovacuometer (Comercial Médica, Brazil) through a mouthpiece to the volunteer, who was in a seated position, resting, and using a nasal clip, according to the Brazilian guidelines for measuring maximum static respiratory pressures. We determined maximal inspiratory pressure after maximal inspiratory effort from functional residual capacity. Likewise, we calculated maximal expiratory pressure after maximal expiratory effort starting from the total lung capacity. The values selected to define maximum respiratory pressures were those obtained in the first second after the peak pressure. Researchers performed the maneuvers with an interval of 30 s and considered the highest value of three measurements ^15^. Predicted values by age and sex were calculated by Costa *et al.*^16^.

#### Peripheral muscle strength

We assessed peripheral muscle strength using a handgrip dynamometer (Saehan Corporation, DSH5001, Korea). Personnel instructed the subject to sit on a chair without support for the arms, with shoulders positioned close to the trunk, elbows flexed at 90°, and forearms in a neutral position. The patient squeezed the dynamometer as hard as possible for five seconds. The test was performed three times for both upper limbs, and the mean between measurements for each limb was taken as the final value. We set a rest time of two minutes between each repetition on the same limb ^17^. The predicted values by age and sex were based on the Schlüsse *et al.* study ^18^.

### Statistical analyses

Data were analyzed using Prism 6.0 software (GraphPad, USA). The Shapiro-Wilk test was used to determine the data distribution. We presented parametric data as a mean ± standard deviation and categorical data as absolute values and occurrence percentages. We applied the unpaired Student t test for comparisons between two independent groups and for comparisons between actual and predicted values, we used the paired Student t test. Pearson's correlation coefficient was used for parametric data and Spearman's correlation coefficient for non-parametric data. We select a significance threshold based on a p value less than 0.05 for all analyses.

### Ethical aspects of the research

The Human Research Ethics Committee of the João de Barros Barreto University Hospital approved this study (certificate number: 1.426.762/2016). All participants signed the informed consent prior to participation. We followed the Strengthening the Reporting of Observational Studies in Epidemiology (STROBE statement) ^19^.

## Results

**Figure 1:**
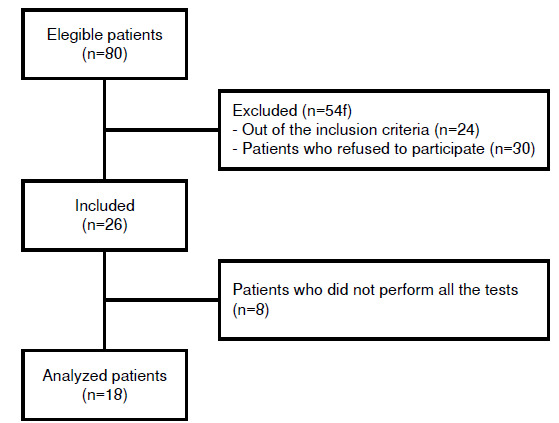
Flow chart of sample distribution

Over the study period, we screened 80 patients with Chagas disease. Of these, 18 met the study inclusion criteria, were diagnosed with acute or chronic Chagas disease, and have been followed-up at the Multidisciplinary Laboratory of Chagas Disease of the João de Barros Barreto University Hospital (figure 1).

Most patients with acute or chronic Chagas disease came from the interior of the State of Pará, with ages ranging from 20 to 60 years and generally presenting a low education level. Several patients presented one or more arrhythmia and/or dyspnea-related complaints (table 1). Additionally, all participants in the acute phase of the disease were undergoing pharmacological treatment with benznidazole.

**Table 1:** Demographic and clinical characteristics of patients with acute and chronic Chagas disease

Variables	Acute Chagas disease	Chronic Chagas disease
	n (%)	n (%)
Origin		
Metropolitan Region of Belém	3 (33.3)	0 (0)
Interior of the State of Pará	6 (66.6)	9 (100)
Sex		
Female	5 (55.5)	5 (55.5)
Male	4 (44.4)	4 (44.4)
Age (years)		
20-30	4 (44.4)	2 (22.2)
31-40	2 (22.2)	2 (22.2)
41-50	1 (11.1)	3 (33.3)
51-60	2 (22.2)	2 (22.2)
Schooling (years)		
< 8	7 (77.7)	4 (44.4)
≥ 8	1 (11.1)	1 (11.1)
Unknown	1 (11.1)	4 (44.4)
Main symptoms		
Arrhythmia	2 (22.2)	1 (11.1)
Dyspnea	3 (33.3)	4 (44.4)
Arrhythmia + Dyspnea	2 (22.2)	2 (22.2)
No symptoms	2 (22.2%)	2 (22.2)
Comorbidities		
SAH	0 (0)	2 (22.2)
Diabetes	0 (0)	0 (0)
SAH + Diabetes	1 (11.1)	0 (0)
No comorbidities	7 (77.7)	4 (44.4)
No information	1 (11.1)	3 (33.3)

SAH: Systemic arterial hypertension

Schooling: eight years represent elementary and high school completion.

Most of the participants in the acute Chagas disease group were younger than the chronic Chagas disease group, where people aged between 41 and 50 years were more prevalent. We did not find significant correlations between the participants' age range and any of the variables explored in this article. Furthermore, we highlight that although a large number of participants originated from rural areas, this characteristic did not have a significant correlation with the six-minute walk test performance (p = 0.35) or the results of maximal inspiratory pressure (p = 0.20), maximal expiratory pressure (p = 0.41), and peripheral muscle strength (p = 0.30 and p = 0.44), suggesting that volunteers' physical performance was not correlated to their place of origin.

### Functional capacity

**Figure 2:**
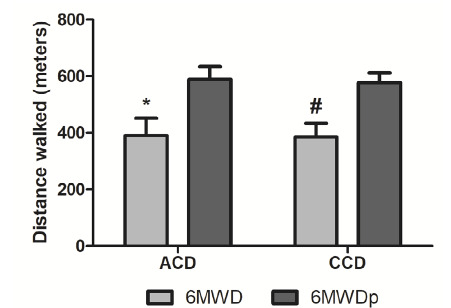
Real and predict functional capacity of acute Chagas disease and chronic Chagas disease groups

Researchers assessed perceived exertion through the Borg scale and oxygen saturation by pulse oximetry. The chronic Chagas disease group presented similar values to those of the acute group on the Borg scale (acute Chagas disease: median = 3, IQR = 1.5-5; chronic Chagas disease: median = 5, IQR = 2-5.5; p = 0.46), and on pulse oximetry (acute Chagas disease = 98% ± 0.9; chronic Chagas disease = 98% ± 1 ; p = 0.46). The actual and predicted distance walked in the six-minute walk test were similar between the acute and chronic Chagas disease groups. However, we observed that both groups walked less distance than the predicted values, with a significant difference between them (p< 0.0001) (figure 2).

We present data mean and standard deviation. Predicted six-minute walk distance (6MWDp) and six-minute walk distance (6MWD). We did not find statistically significant differences between the predicted six-minute walk distance of acute Chagas disease patients versus the same parameter in those with chronic Chagas disease (p = 0.52). The six-minute walk distance of acute versus chronic Chagas disease patients was p = 0.83. Intra group comparisons between actual and predicted six-minute walk distance were statistically significant for acute Chagas disease (p < 0.0001 (*) and chronic Chagas disease (p < 0.0001 (#).

### Cardiovascular performance

When the acute and chronic Chagas disease groups performed the six-minute walk test to evaluate the impact of submaximal exercise on cardiovascular parameters, we did not observe differences between groups for systolic blood pressure at rest or after the six-minute walk test, nor for diastolic blood pressure at rest or after the six-minute walk test.

The heart rate at rest or at the end of the six-minute walk test also did not show significant differences between groups. On the other hand, in the analysis of heart rate within two minutes of the six-minute walk test, we observed an increase in the chronic compared to the acute Chagas disease group. However, at four minutes, there was no statistical difference between the groups. The rate-pressure product presented similar values in the acute and chronic Chagas disease groups, both at rest and after the six-minute walk test. All data are shown in table 2.

**Table 2:** Impact of six-minute walk test on cardiovascular parameters in the acute and chronic Chagas disease groups

Variable	Acute Chagas	Chronic Chagas	p value
	disease	disease	
SBPr (mm Hg)	108 ± 12	112 ± 11	0.47
SBPe (mm Hg)	122 ± 12	124 ± 12	0.76
DBPr (mm Hg)	74 ± 5	79 ± 8	0.17
DBPe (mm Hg)	81 ± 9	81 ± 10	> 0.99
HRr (bpm)	81 ± 12	83 ± 8	0.70
HR^2min^ (bpm)	91 ± 14	103 ± 7	0.04*
HR^4min^ (bpm)	94 ± 12	104 ± 7	0.06
HRe (bpm)	97 ± 12	102 ± 9	0.35
RPPr (mm Hg * bpm)	9 ± 2	9 ± 1	0.95
RPPe (mm Hg * bpm)	12 ± 2	13 ± 1	0.97

Mean, standard deviation and p values for systolic blood pressure (SBP) and diastolic blood pressure (DBP) in mm Hg (r = rest/e= end of six minutes), heart rate (HR) in beats per minute (bpm), measured two and four minutes after the six-minute walk test, and rate pressure product (RPP), in the acute and chronic Chagas disease groups, statistically significant when p < 0.05 (*).

### Respiratory muscle strength and peripheral muscle strength

**Figure 3:**
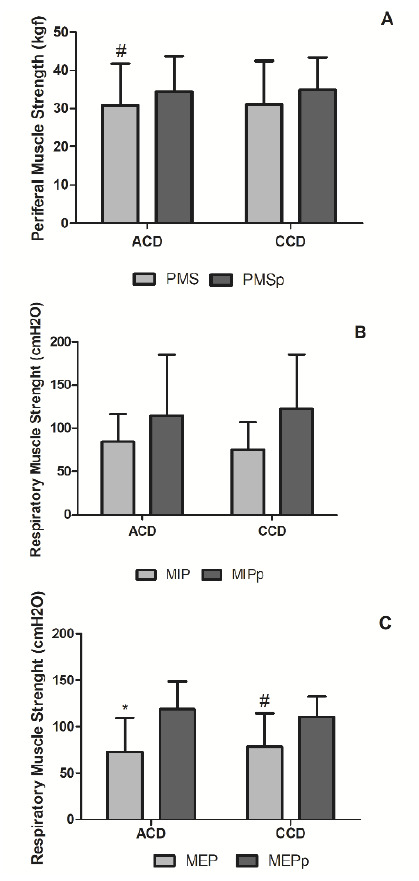
Impact of Chagas disease phase on peripheral and respiratory muscle strength

Peripheral muscle strength, measured with a manual dynamometer on the dominant side in both groups, revealed no statistical difference between groups, or among the predicted values (figure 3A). Comparing actual and predicted values, we observed a reduction only in the acute Chagas disease group.

We estimated the mean, standard deviation, and p values for the variables peripheral muscle strength (PMS), predicted peripheral muscle strength (PMSp) represented as kgF, maximal inspiratory pressure (MIP), predicted maximum inspiratory pressure (MIPp), maximal expiratory pressure (MEP), and predicted maximal expiratory pressure (MEPp) represented as cmH2O. Intra-group comparisons in acute Chagas disease showed statistically significant differences between actual and predicted peripheral muscle strength (p = 0.02 #), and actual and predicted maximal expiratory pressure (p=0.005 *). Chronic Chagas disease presented significant differences only for actual versus predicted maximal expiratory pressure (p = 0.02 #).

We estimated actual and predicted values for inspiratory and expiratory muscle strength by maximum inspiratory pressure and maximum expiratory pressure (figure 3B-C). The authors did not observe differences between groups in actual and predicted maximum expiratory pressure or actual and predicted maximum inspiratory pressure. On the other hand, when comparing actual and predicted values of maximum expiratory pressure, observed ones were lower than expected in both groups, whereas the maximum inspiratory pressure showed no difference between actual and predicted values.

We showed the associations between functional capacity and peripheral and respiratory muscle strength for the acute and chronic Chagas disease groups in table 3. In the acute Chagas disease group, we found a moderate positive correlation between the sixminute walk test and peripheral muscle strength, between maximum expiratory pressure and maximum inspiratory pressure, and between the six-minute walk test and maximum inspiratory pressure. In the chronic Chagas disease group, we observed a moderate correlation only between peripheral muscle strength and maximum inspiratory pressure.

**Table 3:** Correlations among the six-minute walk test, dynamometry (peripheral muscle strength) and manovacuometry (rate-pressure product) in acute and chronic Chagas disease groups

Variables	PMS	MEP MIP	RPP
Acute Chagas disease			
6MWT	0.7860^a^	0.5128 0.7747^b^	-0.5769
PMS	1	0.3690 0.5894	-0.3329
MEP	-	1 0.7974^c^	0.1086
MIP	-	-1	-0.3589
Chronic Chagas disease			
6MWT	0.2071	0.4874 0.5439	0.3363
PMS	1	0.5836 0.7288^d^	-0.2405
MEP	-	1 0.4268	-0.1535
MIP	-	- 1	-0.1004

Pearson's or Spearman's coefficients obtained after correlation analysis between the six-minute walk test (6MWT), peripheral muscle strength (PMS), maximal expiratory pressure (MEP), maximal inspiratory pressure (MEP), and rate-pressure product (RPP). Significant correlations: (a) p = 0.0120; (b) p = 0.0142; (c) p = 0.0100; (d) p =0.0259

## Discussion

In the present study, we observed no differences between acute and chronic Chagas disease groups concerning functional capacity and peripheral or respiratory muscle strength. Patients presented similar sociodemographic profiles, and dyspnea alone or associated with arrhythmia was the main clinical complaint. Similar to our findings, a previous study compared respiratory muscle strength and functional capacity in two groups of patients with chronic Chagas disease and found no differences in these parameters. Furthermore, another study compared respiratory function and respiratory muscle strength of patients with chagasic cardiomyopathy and patients with heart failure due to other etiologies and did not find any differences in these parameters ^15^^,^^20^.

In the current study, we found a lower tolerance to exercise in comparison to reference values in both the acute Chagas disease and chronic Chagas disease groups evaluated through the six-minute walk test. Concerning respiratory muscle strength, when comparing actual and predicted values, a lower-than-expected value was observed in both groups regarding maximum expiratory pressure. Maximum inspiratory pressure did not show a difference. Moreover, when we compared actual to predicted values, only the acute Chagas disease group showed reduced peripheral muscle strength.

Several studies have shown that the function and structure of skeletal muscle are altered in patients with Chagas disease and heart failure ^21^^,^^22^. Montes de Oca and colleagues ^23^ evaluated metabolic and structural characteristics of peripheral muscles in Chagas disease patients and whether they were related to exercise performance. The authors showed a reduction in type I muscle fibers and a proportional increase in type IIb fibers. Thus, there seems to be an oxidative capacity decrease and anaerobic metabolism shifts in the skeletal muscle of Chagas disease patients ^23^. These results indicate that limited peripheral muscle performance may be due to changes in their enzymatic activity and oxygen supply during exercise.

Another interesting finding of our investigation is the correlation between the applied tests. Our group observed a strong correlation between the distance covered in the sixminute walk test and the variables of peripheral muscle strength and maximum inspiratory pressure, and also among maximum inspiratory pressure and maximum expiratory pressure in the acute Chagas disease group. However, in the chronic Chagas disease group, the only significant correlation was between functional performance in peripheral muscle strength and the inspiratory muscle strength indicator (maximum inspiratory pressure), absent in the acute Chagas disease group. These results demonstrate an association between a deficit in functional performance and muscle strength decrease in patients with Chagas disease during the acute phase. Nonetheless, we should consider the multifactorial nature of general deconditioning and the low motivation for exercise.

Respiratory muscle function may be affected by heart-related diseases, in which patients may experience weakness and respiratory muscle failure. Studies indicate that high levels of circulating pro-inflammatory chemokines can cause respiratory and limb weakness, dyspnea, and reduced physical activity in Chagas disease patients ^24^. Montes de Oca *et al.*^23^^)^ demonstrated increased glycolytic and decreased oxidative capacity of peripheral muscles in Chagas disease patients, which may be related to an autoimmune process triggered by the presence of the parasite. This autoimmunity is considered a pathological mechanism for heart and muscle injuries, which can directly affect performance in functional tests. Many studies have found maximum inspiratory pressure as the principal variable affected in patients with Chagas disease or heart failure due to other etiologies. Maximum expiratory pressure does not change ^8^^,^^16^^,^^25^ in contrast with our findings since maximum expiratory pressure was below expected in both groups.

Maximum inspiratory pressure is an important parameter representing the respiratory effort required for daily activities. In addition, it is an independent prognostic predictor relevant to risk stratification for cardiopulmonary impairments ^25^^,^^26^. Although we found reduced actual values of maximum inspiratory pressure, they are still within the predicted normal clinical range, and the correlation found with peripheral force may serve as an indicator.

Forgiarini *et al.* identified respiratory muscle weakness in patients with heart failure ^27^ and detected a significant decrease in maximum inspiratory pressure. Hammond *et al.* demonstrated the presence of respiratory muscle weakness in patients with heart disease, with reduced blood flow to respiratory muscles, leading to generalized muscle atrophy. Moreover, the skeletal muscles of patients with heart failure present a decrease in the diameter of type I and II fibers ^8^. Although manual peripheral force and maximum inspiratory pressure remained constant, the six-minute walk distance was lower than expected in both groups, suggesting cardiorespiratory and musculoskeletal implications in other muscle groups not evaluated, this being a limitation of our study. This reasoning would also help to explain the correlation between the six-minute walk distance and maximum inspiratory pressure variables in the acute Chagas disease group. Chagas diseasetriggered changes can lead to poor exercise performance and fatigue after minimal effort, interfering with functional capacity and inspiratory force.

Regarding vital signs obtained in the six-minute walk test, there was a significant increase in heart rate in the second minute of physical effort in the chronic versus the acute Chagas disease group, reaching maximum heart rate early. We noticed a higher variation in the acute group's resting heart rate, indicating that the maximal functional capacity of Chagas patients may be limited by a deterioration of cardiovascular fitness ^15^.

Another variable evaluated was the relation between the heart rate and systolic blood pressure, termed rate-pressure product, a reliable indicator of oxygen demand by the myocardium and is widely used clinically during rest or physical exertion ^28^. According to previous studies, changes in aerobic fitness are associated with changes in cardiovascular function, like myocardial contractility increase, leading to increased heart rate and blood pressure, thus augmenting cardiac workload and rate-pressure product ^28^^-^^30^. In our study, although we did not find a statistical difference, in the acute Chagas disease group, we observed a correlation between the six-minute walk test and rate-pressure product, suggesting that the acute Chagas group may demand more oxygen to the myocardium to cover a shorter distance.

It is possible that, in addition to cardiac abnormalities, changes in peripheral musculature may contribute to reduced exercise capacity in these individuals ^31^. Musculoskeletal abnormalities in patients with cardiopathies, such as loss of muscle mass, may contribute to exercise intolerance, reducing functional capacity to execute the six-minute walk test. As a main limitation of this study, the six-minute walk test is selfpaced and, therefore, subject to participant motivation. In addition, many of the possible mechanisms suggested can also be attributed to deconditioning and are unlikely to be the cause of lower-than-predicted exercise tolerance in acute Chagas disease.

As previously verified, the results interpretation of this study may suggest that the manifestations observed in the participants possibly derive from both the presence of chronic heart failure and the specific Chagas etiology. Over time, the clinical complexity of Chagas disease can result in chronic cardiac complications, including heart failure. The multifactorial nature of heart failure, influenced by factors such as the progression of Chagas disease, pre-existing conditions, and other cardiovascular risk factors, justify consideration of both possibilities when interpreting the results ^32^.

The present study has limitations, like the small sample we used to obtain our results. Then, larger populations and prospective studies are needed to confirm these findings. Second, the cross-sectional design does not enable causal factors identification of functional changes in Chagas disease patients. However, these findings can broaden knowledge regarding functional characteristics in the different stages of Chagas disease. Third, to stratify the volunteers into two groups, we consulted the hospital records of all volunteers and noticed some essential information missing, such as more details about the onset of the infection by the parasite, limiting the exploration of more comparisons and associations. Furthermore, a substantial limitation of this study lies in the lack of treatment follow-up, especially those in the chronic phase.

The lack of information about pharmacological treatments can compromise results interpretation since specific medications can significantly influence the disease progression and the developed symptoms. The absence of these data limits generalization and highlights the importance of future research incorporating detailed analysis of participants' therapies to enrich understanding of the obtained results at different stages of the disease.

We conclude that Chagas has unique clinical and functional characteristics in each phase of the disease. Both acute and chronic Chagas disease patients presented a belowexpected performance for the six-minute walk test and maximum expiratory pressure, requiring preventive actions to avoid more severe functional losses, such as those affecting autonomy. Early heart rate increase during exercise and its variation in the chronic Chagas disease group suggests a higher myocardial overload and, consequently, the need for additional care in cardiorespiratory training.

Based on these findings, we encourage the development of new research to prevent further injuries, and provide functional improvements to Chagas disease patients, and also to create complementary strategies to the conventional treatment in clinical management, considering evaluation and training of peripheral and respiratory muscles for improvement of symptoms and prognosis.
